# Uptake of self-management education programmes for people with type 2 diabetes in primary care through the embedding package: a cluster randomised control trial and ethnographic study

**DOI:** 10.1186/s12875-024-02372-x

**Published:** 2024-04-25

**Authors:** Melanie J Davies, Danielle H Bodicoat, Alan Brennan, Simon Dixon, Helen Eborall, Agnieszka Glab, Laura J Gray, Michelle Hadjiconstantinou, Lisa Huddlestone, Nicky Hudson, Anju Keetharuth, Kamlesh Khunti, Graham Martin, Alison Northern, Rebecca Pritchard, Sally Schreder, Jane Speight, Jackie Sturt, Jessica Turner

**Affiliations:** 1 Leicester Diabetes Centre, University Hospitals of Leicester NHS Trust, Leicester General Hospital, Gwendolen Road, Leicester, LE5 4PW UK; 2 Diabetes Research Centre, University of Leicester, Gwendolen Road, Leicester, LE5 4PW UK; 3 NIHR Leicester Biomedical Research Centre, Leicester General Hospital, Gwendolen Road, Leicester, LE5 4PW UK; 4 Independent researcher, Leicester, LE7 3SX UK; 5 School of Health and Related Research, University of Sheffield, Regent Court (ScHARR), 30 Regent Street, Sheffield, S1 4DA UK; 6 Usher Institute, Old Medical School, The University of Edinburgh, Teviot Place, Edinburgh, EH8 9AG UK; 7 Department of Population Health Sciences, George Davies Centre, University of Leicester, University Road, Leicester, LE1 7RH UK; 8 Department of Health Sciences, University of York, Seebohm Rowntree Building, Heslington, YO10 5DD York UK; 9 School of Applied Health Sciences, De Montfort University, The Gateway, Leicester, LE1 9BH UK; 10 THIS Institute, University of Cambridge, Clifford Allbutt Building, Cambridge Biomedical Campus, Cambridge, CB2 0AH UK; 11 The Australian Centre for Behavioural Research in Diabetes, 570 Elizabeth Street, Melbourne, VIC 3000 Australia; 12 School of Psychology, Deakin University, Geelong VIC, 3220 Australia; 13 Florence Nightingale Faculty of Nursing, Midwifery and Palliative Care, King’s College London, Strand, London, WC2R 2LS UK

**Keywords:** Implementation, Primary care, Self-management education, Structured education, Type 2 diabetes mellitus

## Abstract

**Background:**

Self-management education programmes are cost-effective in helping people with type 2 diabetes manage their diabetes, but referral and attendance rates are low. This study reports on the effectiveness of the Embedding Package, a programme designed to increase type 2 diabetes self-management programme attendance in primary care.

**Methods:**

Using a cluster randomised design, 66 practices were randomised to: (1) a wait-list group that provided usual care for nine months before receiving the Embedding Package for nine months, or (2) an immediate group that received the Embedding Package for 18 months. ‘Embedders’ supported practices and self-management programme providers to embed programme referral into routine practice, and an online ‘toolkit’ contained embedding support resources. Patient-level HbA1c (primary outcome), programme referral and attendance data, and clinical data from 92,977 patients with type 2 diabetes were collected at baseline (months − 3–0), step one (months 1–9), step 2 (months 10–18), and 12 months post-intervention. An integrated ethnographic study including observations, interviews, and document analysis was conducted using interpretive thematic analysis and Normalisation Process Theory.

**Results:**

No significant difference was found in HbA1c between intervention and control conditions (adjusted mean difference [95% confidence interval]: -0.10 [-0.38, 0.18] mmol/mol; -0.01 [-0.03, 0.02] %). Statistically but not clinically significantly lower levels of HbA1c were found in people of ethnic minority groups compared with non-ethnic minority groups during the intervention condition (-0.64 [-1.08, -0.20] mmol/mol; -0.06% [-0.10, -0.02], *p* = 0.004), but not greater self-management programme attendance. Twelve months post-intervention data showed statistically but not clinically significantly lower HbA1c (-0.56 [95% confidence interval: -0.71, -0.42] mmol/mol; -0.05 [-0.06, -0.04] %; *p* < 0.001), and higher self-management programme attendance (adjusted odds ratio: 1.13; 95% confidence interval: 1.02, 1.25; *p* = 0.017) during intervention conditions. Themes identified through the ethnographic study included challenges for Embedders in making and sustaining contact with practices and providers, and around practices’ interactions with the toolkit.

**Conclusions:**

Barriers to implementing the Embedding Package may have compromised its effectiveness. Statistically but not clinically significantly improved HbA1c among ethnic minority groups and in longer-term follow-up suggest that future research exploring methods of embedding diabetes self-management programmes into routine care is warranted.

**Trial registration:**

ISRCTN23474120, registered 05/04/2018.

**Supplementary Information:**

The online version contains supplementary material available at 10.1186/s12875-024-02372-x.

## Background

More than 3.9 million people in the United Kingdom (UK) live with type 2 diabetes mellitus (T2DM) [1]. International guidelines recommend that pharmacological interventions for managing T2DM need to be supplemented with lifestyle and self-care behaviours for effective glucose management [2], with evidence demonstrating the effectiveness of structured self-management education (SSME) programmes to facilitate the development and maintenance of healthy lifestyle choices [3–8]. These programmes usually feature education, professional support, and peer support as a cost-effective means of improving outcomes for people with T2DM including glycaemic and clinical indicators, perceived wellbeing, and risk behaviours [3–8], and are recommended by the UK National Institute for Health and Care Excellence (NICE), American Diabetes Association, and European Association for the Study of Diabetes for supporting people with T2DM [9, 10].

Prior to structural changes to National Health Service (NHS) commissioning and the introduction of Integrated Care Systems in England, and at the time that the current study was conceived, SSME programmes were selected and delivered by local provider organisations commissioned by a clinical commissioning group (CCG). A referral to the local SSME programme from a primary care provider was usually needed, although some areas permitted self-referral. The inclusion of a referral indicator linked to financial rewards associated with performance and patient outcomes in the NHS Quality and Outcomes Framework [11] increased the rate of referral to SSME within 12 months of T2DM diagnosis from 47% in 2013 to 75% in 2021. Attendance rates are generally low, however, and are estimated to range from 11% (derived from primary/care area data) [12] to 49% (derived from self-report data) [13].

Low rates of referral to and participation in SSME programmes have previously been attributed to a range of factors, including lack of knowledge about the programmes, misconceptions about the cost or effectiveness of SSME programmes, insufficient infrastructure to support management and referral, and lack of consideration of barriers to patient access [14]. Furthermore, attendance and effectiveness data were not routinely collected, limiting evaluation opportunities.

Within this context, an intervention (the ‘Embedding Package’) was designed to improve referral to, and uptake of, SSME programmes by supporting practices to embed SSME referral within routine primary care. The pilot study [15], protocol paper [16], and intervention development paper [17] have been published elsewhere. This paper reports the primary results from the RCT and the ethnographic process evaluation that was conducted to evaluate the effectiveness of the Embedding Package.

## Methods

### Intervention (embedding package)

The development work identified key components of an intervention to embed SSME into primary care. These comprised: a clear marketing strategy for T2DM self-management education programmes; user-friendly and effective referral pathway; new/amended professional roles; and a toolkit of resources [17]. The Embedding Package therefore delivered these components through a new role (‘Embedder’) and an online portal with a range of supporting resources (‘toolkit’).

The toolkit was a password-protected, web-based portal for anyone involved in implementing SSME, including commissioners, providers, and primary care staff, to help them develop embedding strategies. The toolkit comprised three sections with a range of tools, guidance, and sample resources, including how-to guides for activities such as needs assessment and programme adaptation; tools for developing patient-facing marketing and communication materials; and activities to evaluate and strengthen referral pathways and processes.

Two Embedders formed part of the intervention. The role required thorough knowledge about the benefits of SSME, strong communication skills, experience in building strong working relationships, and ability to implement toolkit content. Embedders were employed to promote toolkit use via in-person meetings, email communications, telephone calls, and attendance at health fairs with stakeholders. One Embedder focussed on SSME providers, whilst the other focussed on referring practices. Additionally, Embedders encouraged the introduction and designing of materials specific to general practice and SSME provider needs, working with providers/practices to ensure that localised resources were fit-for-purpose before organisation of production and distribution. This included organising, designing, and delivering presentations at local faith centres to begin discussions about improving access to SSME for ethnic minority populations.

Initially, an Embedder held a face-to-face toolkit action planning meeting with a provider representative to determine which toolkit elements could be implemented. The Embedder then circulated this action plan, including assigning tasks to relevant personnel. Review meetings were scheduled to discuss progress and further tailor the intervention for each locality. Actions relating to practices were disseminated by the Embedder to practice staff and the Embedder presented the toolkit in face-to-face meetings with each practice.

### Study design

**Fig. 1:**
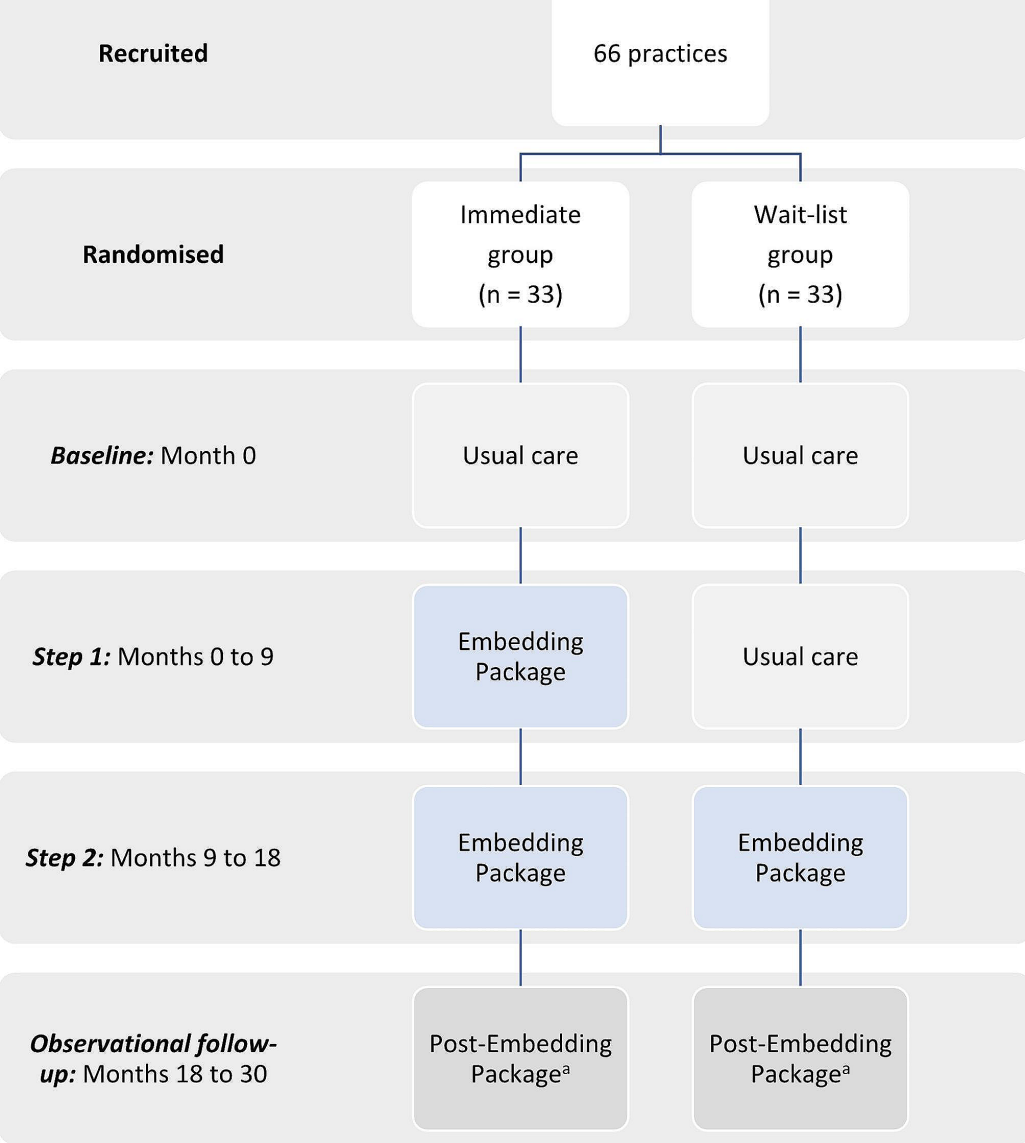
Stepwise Implementation Plan. ^a^ Embedding Package not actively implemented by study team, but available to practices/providers for continued use without the study-provided Embedder

Using a wait-list cluster randomised design with practice-level randomisation, the Embedding Package intervention was compared with the control condition of usual care (practices following their usual SSME programme referral procedures). Informed consent for practice participation was obtained from the practice manager. Implementation and data collection occurred over a stepped timeframe (see Fig. 1) using a wedged stepwise approach. At step 0, baseline data were gathered for three months from all participating practices (months − 3 to 0; baseline) prior to randomisation by an independent statistician to one of two groups (1:1 stratified by CCG). The first group (immediate group) received access to the Embedding Package during step one (months 1–9) and step two (months 10–18), and the second group (wait-list group) received access to the Embedding Package during step two only (months 10–18). The intention was to recruit all practices and then randomise them to groups, but this was not possible due to delays in recruitment, so randomisation was staggered.

Although the trial was implemented over the intended timeframe, the COVID-19 pandemic declared in March 2020 led to restrictions in movement, lockdowns, and redeployment of health care and research staff that continued until the trial concluded in August 2020. Embedding activities ceased in March 2020, as the National Institute for Health Research (the study funder) paused non-COVID-19-related research and redeployed trial staff to COVID-19 research activities. Practices and providers could still access the toolkit during this period. Changes to the protocol were planned by the research team and approved by the study sponsor and funder.

To evaluate the sustainability and effectiveness of the intervention over a longer period, the 18-month implementation period was followed by a 12-month observational period. During this time, the study team did not reinforce the intervention or engage in Embedder activities, but practices and providers continued to have access to the Embedding Package (apart from the study-provided Embedder) and could use it if they chose.

### Sample size

The recruitment target was 66 practices. This target allowed for a 10% drop-out rate from the calculated sample size of 58 practices, which would provide 90% power to detect a 1.1 mmol/mol (0.1%) population-level difference in HbA1c (standard deviation = 16.4 mmol/mol [18]), an ICC of 0.05, and HbA1c measurement at baseline, step 1, and step 2 [19]. Assessment of sensitivity to changes in average cluster size found 80% power to detect a difference of 0.7 mmol/mol (0.06%), andif average cluster size exceeded 174 there would be 80% power to detect a 1.1 mmol/mol (0.1%) diffrence.

### Data collection

Analytics data accessible from the toolkit website included a count of daily website and page views, active accounts, and average time spent on a page.

Clinical data from primary care records for all patients with a T2DM diagnosis attending one of the participating practices were extracted retrospectively at the end of the study for baseline, step 1, step 2, and the observational follow-up period. Where multiple data points were available within one of these timeframes, the most recent measurement was used.

Where an SSME course involved multiple sessions, attendance was defined as attending one or more session in accordance with the national criteria for structured education [20]. As most people only attend SSME programmes once, any attendance prior to the trial or during step one in the wait-list group would not be captured; therefore, while acknowledging that other factors may influence clinical outcomes, patient-level HbA1c was used as a proxy for SSME attendance and was the primary outcome for the trial, as attending an SSME programme was expected to result in lower HbA1c [3]. The main secondary outcomes were SSME referrals and attendance data (yes/no). Other secondary outcomes were weight, body mass index, cholesterol and blood pressure indicators, cardiovascular risk score, smoking status, hospital admissions, and use of glucose or blood pressure lowering medication.

### Statistical analysis

Intention-To-Treat (ITT practices) were those that agreed to participate and provided baseline data. Complete-case practices were ITT practices that did not withdraw from the study or miss a data extraction.

Descriptive analyses were used to summarise patient data according to randomisation group at practice level (immediate vs. wait-list). Intervention and control data were based on the step at which the intervention was implemented for each group. Baseline data served as control data for the immediate group, which received the intervention during steps one and two, and data collected at baseline and step one served as control data for the wait-list group, which received the intervention in step two. Data regarding patient referral to and attendance at SSME were carried forward to subsequent steps.

Mixed effects linear regression modelling was used to explore differences in HbA1c levels between intervention and control conditions (95% confidence interval). Intervention group, CCG, step (baseline, 0–9 months, 9–18 months), and time (month from overall baseline) were entered as fixed effects, and practices and patients as random (nested) effects. An identity correlation matrix was generated. The primary analysis used multiple imputation for patients with missing HbA1c data (ten rows per patient) using predictive mean matching with sex, age, ethnicity, and baseline HbA1c. Two-sided *p*-values were calculated. Sensitivity analyses were applied to explore the effect of the intervention in subpopulations related to ethnicity, complete-case, attendance at SSME, excluding HbA1c < 47.5 mmol/mol at baseline, and up to the end of February 2020 with interactions fitted to evaluate differences. The threshold for clinically significant change in HbA1c at the population level was 1.1 mmol/mol (0.1%) (standard deviation = 16.4 mmol/mol) which may reflect a 2.1% decrease in micro and macrovascular events [19].

Mixed logistic regression models were used to analyse SSME referral and SSME attendance using the same process. Multiple imputation was not possible due to convergence, so where binary values were missing the baseline value was used. To evaluate effectiveness of the intervention over time, analyses exploring HbA1c, SSME referral, and SSME attendance were repeated with inclusion of the data collected 12 months after the end of the intervention.

Model assumptions were checked prior to analysis. Analyses were performed in Stata v17.0 (StataCorp LLC, Texas).

### Ethnographic study

An ethnographic study was conducted to understand the context and implementation process of the Embedding Package within primary care practices, associated CCGs, and SSME providers. The original design had involved a series of face-to-face interviews and observations in 12 of the 66 primary care practices and associated CCGs and SSME providers. The restrictions on physical co-location to reduce the spread of COVID-19 prevented face to face interviews and observations from taking place, requiring changes to the design that were planned by the research team and approved by the study sponsor. Due to limited engagement from practices and providers, data were gathered from Embedder-generated study documents; emails between Embedders, practices, and providers; a time tracking database that Embedders used to track Embedding activities; interviews; and observation of meetings between Embedders and practices.

Interpretive thematic analysis and theoretical insights from Normalisation Process Theory (NPT) were applied to the data in four stages.

1. Emails were grouped by CCG locality and stakeholder group, and content analysis was conducted [21].
2. Interviews were coded using interpretive thematic analysis [22], generating the main themes.
3. Tracker data and study documents were analysed using content analysis, drawing on sensitising categories from the interview data analysis.
4. Data from all sources were integrated into one narrative summary, informed by the use of NPT [23], generating the final thematic structure.

Observations of meetings between Embedders and practices provided background understanding that informed analyses.

## Results

### Population

**Fig. 2:**
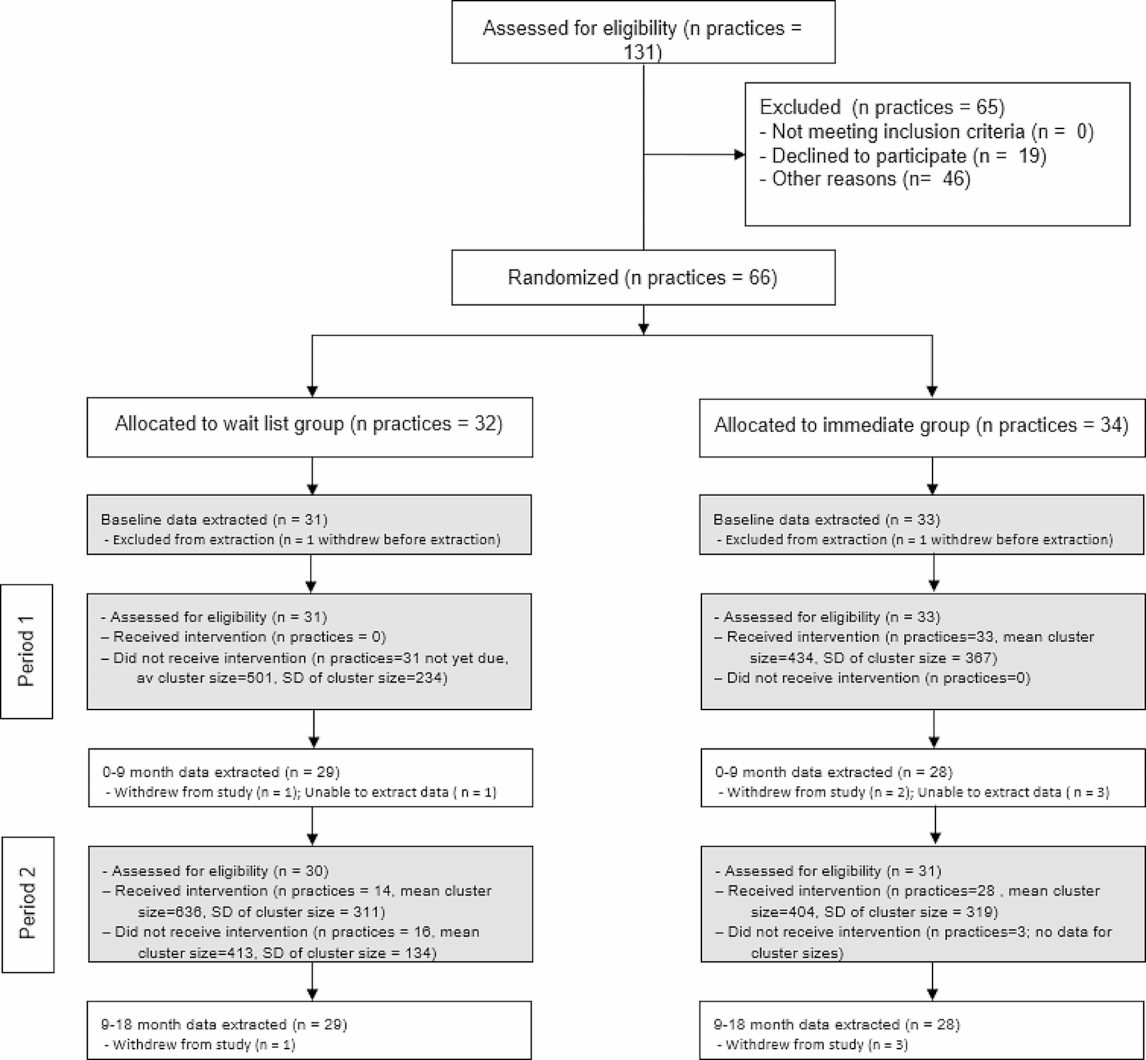
CONSORT diagram^a^. ^a^ Receiving the intervention was defined as participating in at least one aspect of the intervention. *Note: ‘Other reasons’ for exclusion included non-response (42); response after recruitment closed* (1); *interested but lacked current capacity (3)*

Out of 64 ITT practices (31 wait-list; 33 immediate), there were 57 complete-case practices (29 wait-list; 28 immediate) from 10 CCGs across East Midlands, Thames Valley and South Midlands, and Yorkshire and Humber (see Fig. 2). Of the eight SSME providers used by participating practices, one declined to participate, and one withdrew consent. Four SSME programmes were used: DESMOND, Diabetes 2gether/Diabetes 4ward, Spotlight, and Xpert Health. DESMOND was the most frequently used programme for both intervention groups (61.3% wait-list, 57.6% immediate).

At baseline, there were 29 849 eligible people with T2DM registered with ITT practices (15 527 wait-list; 14 322 immediate). Wait-list practices had an average of 501 (standard deviation = 234) patients with T2DM, and immediate practices an average of 434 (standard deviation = 367). Demographic and clinical characteristics at baseline were similar between wait-list and immediate groups (Table 1), with the majority of patients with T2DM being of white ethnicity (75% wait-list; 67% immediate), male (57% wait-list; 58% immediate), and average age of 66–67 years (wait-list = 67 (interquartile range [IQR]: 56,76); immediate = 66 (IQR: 56, 75)).

**Table 1:** Descriptive baseline characteristics of patients with type 2 diabetes mellitus registered at participating practices

		Wait-list (*n* = 15,527)		Immediate (*n* = 14,322)	
Characteristic	% missing	Median	IQR	Median	IQR
Age, years	0.0	67	56, 76	66	56, 75
HbA1c, mmol/mol	8.2	53	47, 63	53	47, 63
HbA1c, %	8.2	7.0	6.5, 7.9	7.0	6.5, 7.9
Time from HbA1c measurement to baseline, weeks	8.2	14.1	6.6, 24.1	14.6	7.1, 24.1
		**N**	**%**	**N**	**%**
Sex^a^					
Men		8773	56.5	8255	57.6
Women		6754	43.5	6067	42.4
Ethnicity					
White		11 629	74.9	9647	67.4
South Asian		927	6.0	1002	7.0
Black		442	2.9	603	4.2
Other		554	3.6	558	3.9
Missing		1975	12.7	2512	17.5
SSME status^a^					
Not referred		2989	19.3	5951	41.6
Referred, not attended		2544	16.4	2879	20.1
No referral, but attended^b^		3188	20.5	1803	12.6
Attended		6806	43.8	3689	25.8

Abbreviations: IQR, Interquartile Range; SSME, Structured self-management education

^a^ No missing data

^b^ Likely self-referral

A higher proportion of patients from wait-list practices had already attended SSME at baseline (64%) compared with patients in the immediate group (38%), with non-referral more common amongst immediate patients (42%) than wait-list patients (19%). Wait-list patients were slightly more likely to have been referred but not attended (20%) than immediate patients (16%).

### Toolkit usage

Of 88 live toolkit accounts, generic accounts accessible to more than one person were assigned to 68 practices (77.3%). Eighteen providers (20.5%), and 2 commissioners (2.3%) were assigned accounts. The toolkit yielded an average of 19 page views per day, including views by study staff that could not be extracted from the data. Between 16 and 267 s were spent viewing each page (mean = 106.9, standard deviation = 66.78).

### Primary outcome

After controlling for clustering and time effects, the primary outcome of HbA1c was not significantly different between the intervention and control conditions in the main analyses (adjusted mean difference = -0.10 (95% CI = -0.38, 0.18) mmol/mol or 0.01% (95% CI = − 0.03, 0.02); *p* = 0.503) or the majority of the sensitivity analyses (Table 2). The treatment effect for ethnic minority groups was statistically but not clinically significantly different from that found for non-minority groups with an adjusted mean difference of -0.64 (95% CI = -1.08, -0.20) mmol/mol or -0.06% (95% CI = -0.10, -0.02); *p* = 0.004).

**Table 2:** Sensitivity analyses for the primary outcome (HbA1c in mmol/mol) in different populations

	Control period	Intervention period	Control period	Intervention period	Mean difference (95% confidence interval)	*P*-value
Population	Number of observations		Mean (standard deviation)			
*Ethnicity*						
White	29 398	23 521	57.18 (16.03)	57.93 (16.42)	-0.02 (-0.33, 0.29)	0.913
Ethnic minority groups	5732	5850	59.13 (17.77)	59.06 (16.98)	-0.64 (-1.08, -0.20)	0.004
Complete-case	36 396	31 601	57.40 (16.19)	58.09 (16.50)	-0.14 (-0.45, 0.16)	0.361
Only those who attended education	22 570	16 792	56.97 (15.76)	57.60 (16.14)	-0.11 (-0.51, 0.29)	0.596
Excluding HbA1c < 47.5 mmol/mol at baseline	29 724	24 962	62.26 (15.83)	61.06 (16.65)	-0.04 (-0.41, 0.33)	0.832
Up to end of February 2020	40 112	10 717	57.37 (16.21)	57.62 (15.80)	0.12 (-0.21, 0.46)	0.477

Note: Missing data were not imputed, except in the ethnicity model which used the primary analysis approach

### Main secondary outcomes

Referral to SSME was significantly lower during intervention conditions compared with control (OR (95% CI) = 0.85 (0.73, 0.99; *p* = 0.038)), but lower attendance at SSME during intervention conditions was not statistically significant (OR (95% CI) = 0.82 (0.66, 1.01; *p* = 0.063)) (Table 3).

**Table 3:** Intervention impact in the intention-to-treat population (64 practices; 35,155 patients; 92,977 observations)

	Complete-cases^a^				Intention-To-Treat^a^	
	Number of observations		Mean (standard deviation)		Mean difference (95% CI)	*P*-value
Outcome	Control (Total = 45,940)	Intervention (Total = 47,037)	Control	Intervention		
HbA1c, mmol/mol^b^	40 112	31 601	57.37 (16.21)	58.09 (16.50)	-0.10 (-0.38, 0.18)	0.503
			**Number (%)**		**Odds ratio** **(95% CI)**	***P-value***
Referred	40 619	39 368	23 237 (57.2%)	21 981 (55.8%)	0.85 (0.73, 0.99)	0.038
Attended	40 619	39 368	23 376 (57.6%)	20 470 (52.0%)	0.82 (0.66, 1.01)	0.063

Abbreviations: CI, Confidence Interval; IQR, Interquartile range

^a^ The intention-to-treat population was used for the analysis with multiple imputation to impute missing values. However, means for the intention-to-treat population could not be generated using multiple imputation, so the complete-cases population was used for the summary statistics. The summary data are crude data and do not account for factors included in the model (imputation of missing data, nested random effects to account for non-independence of data, adjustment for covariates), therefore the crude estimates and model estimates are not directly comparable, which is why some effect sizes are in the opposite direction to the summary data

^b^ Pre-specified primary outcome

### Other secondary outcomes

Other secondary outcomes showed no notable differences (see supplementary materials).

### Effectiveness 12 months post-intervention

To evaluate the sustainability of the intervention over time, combined data from 37,825 patients (wait-list = 20,352; immediate = 17,473) from the 18-month trial and the 12-month follow-up were analysed. When controlling for nesting and time effects, HbA1c was lower during the intervention than control conditions (-0.56 [95% CI = -0.71, -0.42] mmol/mol or -0.05% [-0.06, -0.04]; *p* < 0.001). While no significant difference in SSME referrals was found (OR = 0.97; 95% CI = 0.90, 1.04; *p* = 0.414), there were higher levels of programme attendance (adjusted odds ratio = 1.13; 95% CI = 1.02, 1.25; *p* = 0.017) during intervention than control conditions.

### Ethnographic study

The final data set comprised: 2051 emails between Embedders, practices, and providers organised into conversations where appropriate; 928 entries in the intervention ‘tracker’; 49 study documents; one interview with an SSME provider; and eight debriefing interviews with the two Embedders (Table 4).

**Table 4:** Description of the ethnographic WS3 Sample

Method	Source	N	Detail
***Documentary*** ***analysis***	Study documents	49	Such as action plans, meeting notes, updates, publicity materials, etc.
	Digital comms (emails)	2051	Emails between Embedders, providers, and practices
	Tracker data	1	Database detailing all activity (928 entries)
***Interviews***	Interviews	8	Interviews with Embedding team and provider

Two key themes regarding intervention context and implementation were identified: making and sustaining contact; and practice/provider interaction with the toolkit.

### Making and sustaining contact

Data reflected challenges with initiating and sustaining contact with practices and providers. Although Embedders described positive responses to face-to-face initial meetings, this was followed by delayed or no communication or action: “[This is a] prime example of ‘great when you’re in room, walk away from the room and they forget you exist!’ And then taking four months to get any real reply” (Embedder). 60% of Embedders’ time was spent on email communications and administration. Embedders were proactive with emails, reminders, and ongoing communication, with tracking data suggesting that email follow-up with providers and practices accounted for 13% of tracked Embedder activities. Reasons proposed for poor communication from providers and practices included lack of capacity, staff turnover, poor handover practices, and anxiety about additional workload. While the data suggested that prior contact with key stakeholders in CCG localities could improve reception of the Embedding Package, concerns from practices and providers regarding the distribution of costs and benefits were found across CCG localities: “I think they were very conscious of it not adding to their workload. There [were] lots of questions early on about ‘what does this mean for us?’” (Embedder).

### Interaction with the toolkit

Time required to implement and use the toolkit was a common concern across practices and providers. All data sources reflected a high level of proactivity by Embedders to support practices and providers at a local level, including structuring action plans, localising promotional materials, and drafting publicity materials. Considerable time and effort were spent by Embedders on contextualising interventions to meet local needs, with tracker and interview data reflecting significant efforts to account for unique needs within local contexts. Their efforts were appreciated by practices and providers: “… [they’ve] been very useful at … getting us support with the communication side of things… So [the Embedder] definitely helped” (Provider).

Differences across localities in existing provision of and infrastructure for SSME, including routines for referral, practice-provider relationships, and processes for self-referral, had a substantial impact on referral activities. Although the Embedding Package was designed to account for these differences, Embedders lacked local knowledge to navigate these complexities on their own, and saw limited engagement from local stakeholders. Funding disputes and differing opinions about the role of clinicians to gatekeep access to SSMEs further obstructed Embedding activities: “… in reality, unfortunately all of their diabetes education is coordinated via a call centre hub, and they are not open to the idea of self-referral” (Embedder).

Limitations on staff capacity and existing processes limited interaction with the toolkit, even where providers and practices agreed that the toolkit was a good idea. Seeing merit in the toolkit did not often translate into engagement with it, with Embedders suggesting that changing people’s beliefs and the organisational culture around integrating the toolkit into existing practices and processes “…is going to take a lot more than putting up a few posters” (Embedder).

Embedder activities were curtailed in March 2020 due to the COVID-19 pandemic, increasing challenges in communication with practices and providers. While Embedders noticed positive engagement with social media posts from practices and providers, they did not notice a corresponding increase in engagement with the toolkit.

## Discussion

This study aimed to evaluate the effectiveness of the “Embedding Package”, a complex intervention designed to increase rates of referral to, and attendance at, SSME programmes by people with T2DM [17]. Implementation of the Embedding Package was not associated with significantly decreased levels of HbA1c, the primary outcomes for this study.

The ethnographic study identified multiple barriers to effective implementation of the Embedding Package, with limited engagement of practices and providers creating additional challenges. While practices and providers perceived the toolkit as a good idea, due to concerns about time, workload, and perceived benefit many did not access this resource or integrate it into routine practice, echoing similar themes raised in previous research related to the implementation of toolkits [24] and practice guidelines [25]. Additionally, the Embedding intervention targeted the referral system, meaning that while it advocated for referral to SSME programmes it did not have any direct control over programme effectiveness, which may have impacted both the primary outcome and perceptions of referrers and patients about programme usefulness.

These concerns may also have influenced willingness to engage in communication with the Embedders, with Embedders’ spending around 13% of their time on following up with practices and providers alone. Given the high time burden related to emails and administration, email communication may be an ineffective and inefficient use of Embedders’ time. Workload concerns may also have influenced willingness to participate in ethnographic data collection activities, as reflected by low levels of practice and provider participation.

While stakeholders appreciated the importance of adapting the intervention to local contexts, much of the embedding, localisation, and communication work was performed by Embedders whose role spanned multiple localities. The lack of local stakeholder engagement with adaptation activities limited the extent to which the intervention could be adapted to the local context, aligning with findings in the humanitarian space that emphasise the crucial role of local stakeholder participation in localisation activities [26]. While Embedders were proactive in pursuing engagement with local stakeholders, they encountered challenges due to differences in processes, interorganisational relationships, and opinions about accessibility and gatekeeping of SSME, perceiving that higher-level cultural and organisational change was needed to bring about the intended change.

In addition to limited uptake, Embedding activities also ceased in March 2020 before the end of the trial in August 2020 due to the COVID-19 pandemic, forcing the premature cessation of Embedder activities which may have limited effectiveness of the intervention. It is also possible that the trial design underestimated the length of time required for Embedding activities to result in systemic change.

Structural changes to NHS commissioning that occurred after the commencement of the Embedding Package programme may have impacted on uptake of the programme, as the introduction of Integrated Care Systems [11] led to many localities receiving increased funding to support increased uptake of SSME programmes. Similarities in scope and approach to the Embedding Package may have led to a perception within those localities that the Embedding Package was not relevant to them. Furthermore, the study was impacted by changes to the way that service support cost funding was organised and managed during the trial.

Although the Embedder model sought to avoid placing undue burden on local staff, the perception that it would add to workload significantly impeded engagement with the intervention. Misunderstandings about workload impact and the purpose and value of the intervention, as well as conflicts regarding eligibility and access, impacted negatively on stakeholder engagement, leading Embedders to spend a large proportion of their time pursuing communication with providers and practices and contextualising the intervention to local needs. As Embedders sat within the study team and worked across multiple CCG localities, they were not integrated into care teams and lacked local knowledge. Localisation and engagement work may have been more effective if led by local stakeholders [26], or the Embedder role had been integrated into local-level provider organisations [27], which should be a priority research question for future work in this area. Embedders sought primarily to work with professional stakeholders, such as general practices, rather than directly with patients, with local organisations responsible for leading efforts to engage patients; an alternative approach focusing on patient engagement directly may have resulted in greater uptake.

Among patients with T2DM from ethnic minority groups, exposure to the Embedding Package was associated with slightly reduced levels of HbA1c, but not increased engagement with SSME programmes, suggesting that this improvement was not related to Embedding Package activities. It is possible that the Embedding activities focussed on ethnic minority populations may have influenced this improvement; however, the COVID-19 pandemic disrupted further work in adapting SSME content for cultural appropriateness, which is another crucial factor in increasing uptake of SSME among ethnic minority groups [28]. Given the disproportionate impact of T2DM amongst ethnic minority groups [29] and the compounding impact of COVID-19 on people with T2DM in these underserved populations [30, 31], it is crucial that future efforts to increase uptake of SSME avoid increasing inequalities, and account for factors that can compromise the effectiveness of such initiatives [32]. The Embedding Package did not appear to increase existing inequalities, suggesting that some elements may be useful for exploring how SSME uptake may be increased among underserved populations; for example, the refinement of the digital SSME package MyDESMOND for use amongst South Asian and Black ethnic minority groups using a Community-based Participatory Research model (NIHR205180).

The main secondary outcomes were rates of referral to, and attendance at, SSME programmes. Baseline SSME attendance was higher than expected in both the wait-list and immediate practice groups, with the high baseline attendance in the wait-list group (64%) (part of the control condition) leaving less room for improvement than anticipated. This could partly explain why the Embedding Package trial did not significantly impact upon SSME referral or attendance. Baseline attendance in the immediate group was 38%, similar to self-reported rates of attendance of 49% found in previous research [13]. Together, these estimates suggest that the rate of attendance reported by the National Diabetes Audit (11%) is not capturing the full picture of SSME referral and attendance in the UK [12], perhaps because the National Diabetes Audit data focus only on the 12 months after diagnosis.

When the RCT and 12-month observational follow-up data were combined, HbA1c and SSME attendance were improved in intervention compared with control conditions. The HbA1c difference was not clinically significant based on the minimum clinically important difference used in the RCT sample size calculation. While this may reflect that systemic changes, and subsequent clinical improvements, may require a longer period of time to be realised, factors other than the Embedding Package are likely to have contributed to these changes. Follow-up data collection occurred during the COVID-19 pandemic, which also exerted considerable pressure on primary care. The improvements in HbA1c and SSME attendance contrast with research reporting negative impacts of self-isolation [33] and the COVID-19 pandemic on diabetes self-management [34], but align with research suggesting that COVID-19-avoidant behaviours can improve diabetes self-management [35]. This also suggests that the shift to online T2DM SSME delivery and increased health messaging regarding T2DM as a risk factor for COVID-19 complications [36] are likely to have influenced HbA1c levels and SSME referral and engagement during the pandemic, particularly as online delivery has been found to be an effective means of diabetes self-management programmes [37, 38] and warrants further exploration.

### Limitations

Delays related to the feasibility study [15] reduced the time between feasibility study and RCT commencement, leaving insufficient time to implement a key lesson from the feasibility study. The feasibility study identified that the intervention should be implemented at provider-level, not at practice-level, but redesigning the RCT would have required either an extension to the programme timeframe or further redesign of the programme in order to gain back the time. This presents a substantial limitation, which was compounded by the need to put measures in place to avoid contamination whereby control practices received the intervention early via their SSME provider. Providers were asked to only target practices currently receiving the Embedding Package, which may have reduced engagement by adding complexity to provider activities.

Using HbA1c as a proxy for SSME attendance is also a limitation. While the intervention was intended to improve rates of SSME referral and attendance, using these indicators within a wait-list design introduced limitations because a person who attended SSME in a previous step was often ineligible to attend again under local commissioning rules. Furthermore, these measures are not routinely collected, compromising the validity of these measures as primary outcomes. HbA1c was chosen as a proxy as it was likely to change as a result of exposure to SSME [3], but being indirectly affected by referral to and attendance at SSME it may not be sensitive to change.

Additionally, it is possible that the premature cessation of Embedder activities due to the COVID-19 pandemic may have contributed to the limited effectiveness; this is unlikely, however, given the limited engagement of local stakeholders before the pandemic began.

Finally, statistical modelling presented issues related to missing data, model stability, and goodness of fit for binary variables SSME referral and SSME attendance, meaning that caution should be used in interpreting results.

## Conclusion

The current study suggests that the Embedding Package is likely to have had little effect on HbA1c, SSME referrals and attendance, or other secondary outcomes. Slightly lower HbA1c was found among participants from ethnic minority groups during intervention than control conditions, but this was not accompanied by increased levels of SSME attendance. Additionally, longer term analyses showed some evidence of positive outcomes, suggesting that the study period may not have been long enough to see an impact. Social and practical barriers to effective implementation are likely to have contributed to the limited effectiveness of the programme.

### Electronic supplementary material

Below is the link to the electronic supplementary material.

Supplementary Material 1

## Data Availability

The data are not publicly available as they contain identifiable information.

## Abbreviations

CCG: Clinical Commissioning Group
IQR: Interquartile Range
ITT: Intention-To-Treat
NHS: National Health Service
NICE: National Institute for Health and Care Excellence
RCT: Randomised Controlled Trial
SD: Standard Deviation
SSME: Structured Self-Management Education
T2DM: Type 2 Diabetes Mellitus
UK: United Kingdom
