# Supplementary material for: Uptake of self-management education programmes for people with type 2 diabetes in primary care through the embedding package: a cluster randomised control trial and ethnographic study
Source: BMC Prim Care. 2024 Apr 25;25:136. doi: 10.1186/s12875-024-02372-x (PMC11046789; doi:10.1186/s12875-024-02372-x)
Supplement: Supplementary file 1 — Supplementary Material 1 [file 12875_2024_2372_MOESM1_ESM.docx]

Supplementary materials

**Other secondary outcomes**

Significantly more patients smoked during the intervention condition than the control (OR = 1.18 [95% CI = 1.09, 1.28]; p<0.001; Table 2). Average cholesterol was significantly lower by 0.12 (0.08, 0.15) mmol/l during the intervention than control condition (p<0.001). No significant differences were found for the other clinical outcomes (See Table 2).

**Table S1:** Descriptive baseline characteristics of patients with type 2 diabetes mellitus registered at participating practices (secondary outcomes).

|  |  | **Wait-list (n = 15,527)** | | **Immediate (n = 14,322)** | |
| --- | --- | --- | --- | --- | --- |
| **Characteristic** | **% missing** | **Median** | **IQR** | **Median** | **IQR** |
| Body mass index, kg/m^2^ | 19.0 | 30.5 | 27.0, 35.0 | 30.4 | 26.8, 35.0 |
| Weight, kg | 50.2 | 85.5 | 73.0, 100.0 | 86.0 | 74.0, 100.0 |
| Total cholesterol, mmol/l | 13.0 | 3.5 | 2.7, 4.3 | 3.7 | 3.0, 4.5 |
| LDL cholesterol, mmol/l | 49.5 | 1.9 | 1.4, 2.6 | 1.9 | 1.5, 2.6 |
| HDL cholesterol | 14.2 | 1.2 | 1.0, 1.4 | 1.2 | 1.0, 1.4 |
| Systolic blood pressure, mmHg | 7.7 | 131 | 123, 139 | 131 | 122, 140 |
| Diastolic blood pressure, mmHg | 7.7 | 75 | 70, 80 | 76 | 70, 80 |
| Cardiovascular risk score (QRisk) | 83.9 | 23.4 | 13.7, 34.5 | 22.2 | 12.9, 32.8 |
|  |  | **N** | **%** | **N** | **%** |
| Smoking |  |  |  |  |  |
| Non-smoker |  | 8852 | 57.0 | 7882 | 55.0 |
| Ex-smoker |  | 3214 | 20.7 | 4015 | 28.0 |
| Current smoker |  | 3000 | 19.3 | 2278 | 15.9 |
| Missing |  | 461 | 3.0 | 147 | 1.0 |
| Glucose lowering medication^a^ |  |  |  |  |  |
| No |  | 2958 | 19.1 | 2831 | 19.8 |
| Yes |  | 12,569 | 81.0 | 11,491 | 80.2 |
| Blood pressure lowering medication^a^ |  |  |  |  |  |
| No |  | 4092 | 26.4 | 3943 | 27.5 |
| Yes |  | 11,435 | 73.7 | 10,379 | 72.5 |

Abbreviations: IQR, Interquartile Range; SSME, Structured self-management education.

^a^ No missing data.

**Table S2:** Intervention impact on other secondary outcomes in the intention-to-treat population (64 practices; 35,155 patients; 92,977 observations).

|  | **Complete-cases^a^** | | | | **Intention-To-Treat^a^** | |
| --- | --- | --- | --- | --- | --- | --- |
|  | **Number of observations** | | **Mean (standard deviation)** | | **Mean difference**  **(95% CI)** | **P-value** |
| **Outcome** | **Control**  **(Total=45,940)** | **Intervention**  **(Total=47,037)** | **Control** | **Intervention** |  |  |
| Body mass index, kg/m^2^ | 35,422 | 25,133 | 31.58 (6.72) | 31.68 (6.78) | -0.10 (-0.31, 0.10) | 0.295 |
| Weight, kg | 25,985 | 24,782 | 88.71 (21.68) | 89.93 (21.42) | -0.15 (-0.78, 0.48) | 0.633 |
| Total cholesterol, mmol/l | 37,092 | 26,517 | 3.81 (1.20) | 4.22 (1.10) | -0.12 (-0.15, -0.08) | <0.001 |
| LDL cholesterol, mmol/l | 21,442 | 16,503 | 2.07 (0.88) | 2.08 (0.89) | 0.00 (-0.02, 0.03) | 0.879 |
| HDL cholesterol, mmol/l | 36,553 | 26,170 | 1.22 (0.34) | 1.23 (0.35) | 0.00 (-0.01, 0.01) | 0.674 |
| Systolic blood pressure, mmHg | 40,347 | 29,304 | 131.64 (14.21) | 131.42 (16.13) | -0.30 (-0.72, 0.13) | 0.168 |
| Diastolic blood pressure, mmHg | 40,347 | 29,020 | 74.92 (9.54) | 75.35 (9.52) | -0.02 (-0.29, 0.25) | 0.897 |
| Cardiovascular risk score (QRisk) | 7137 | 3,789 | 24.64 (14.68) | 24.05 (14.58) | -0.01 (-0.38, 0.35) | 0.937 |
|  |  | | **Number (%)** | | **Odds ratio**  **(95% CI)** | **P-value** |
| Current smoker | 45,294 | 46,906 | 7455 (16.5%) | 6460 (13.8%) | 1.18 (1.09, 1.28) | <0.001 |
| Any hospital admissions | 44,249 | 38,798 | 1,916 (4.3%) | 1,393 (3.6%) | 0.91 (0.77, 1.08) | 0.279 |

Abbreviations: CI, Confidence Interval; IQR, Interquartile range.

^a^ The intention-to-treat population was used for the analysis with multiple imputation to impute missing values. However, means for the intention-to-treat population could not be generated using multiple imputation, so the complete-cases population was used for the summary statistics. The summary data are crude data and do not account for factors included in the model (imputation of missing data, nested random effects to account for non-independence of data, adjustment for covariates), therefore the crude estimates and model estimates are not directly comparable, which is why some effect sizes are in the opposite direction to the summary data.
